# Knockout of α-Synuclein Is Associated with Depression-like Behaviors by Altered Excitability of Medial Prefrontal Cortex Neurons in Mice

**DOI:** 10.3390/ijms27125235

**Published:** 2026-06-09

**Authors:** Tong Shang, Yu Zhang, Xueling Zhang, Wei Liang, Yunlin Han, Zhiwei Yang, Ling Zhang, Chuan Qin

**Affiliations:** NHC Key Laboratory of Comparative Medicine, National Center of Technology Innovation for Animal Model, National Human Diseases Animal Model Resource Center, Key Laboratory of Pathogen Infection Prevention and Control (Ministry of Education), Institute of Laboratory Animal Science, Chinese Academy of Medical Sciences & Peking Union Medical College, Beijing, 100730, China; shangtong201503@163.com (T.S.); 90905135@pku.edu.cn (Y.Z.); zhangxueling24@163.com (X.Z.); weil0421@163.com (W.L.); 18510165683@163.com (Y.H.); yangzhiwei@cnilas.pumc.edu.cn (Z.Y.)

**Keywords:** α-synuclein, depression, DREADD, GABAergic neurons

## Abstract

To investigate the neural circuit mechanisms linking the physiological loss of α-synuclein (α-syn) function to depressive-like states, we explored whether constitutive α-syn depletion disrupts the excitation and inhibition balance within the medial prefrontal cortex. We integrated comprehensive behavioral paradigms, whole-cell patch-clamp electrophysiology, and targeted chemogenetics within an α-syn knockout mouse model. Behavioral profiling revealed that α-syn deficiency was accompanied by basal psychomotor hyperactivity and profound stress-coping deficits. Cellular electrophysiology in the medial prefrontal cortex demonstrated a significantly enhanced intrinsic excitability across both GABAergic and glutamatergic populations. Furthermore, targeted chemogenetic suppression of GAD67-positive interneurons partially alleviated the depressive-like phenotype without confounding baseline locomotion. In summary, the physiological depletion of endogenous α-syn is associated with altered stress-coping behaviors and increased mPFC neuronal excitability. Chemogenetic suppression of mPFC GAD67-positive interneurons partially reduces immobility in SYN-KO mice, implicating prefrontal inhibitory circuit dysregulation in these behavioral alterations. These findings underscore the importance of α-syn homeostasis in modulating cortical microcircuits and provide novel insights into the early non-motor manifestations of synucleinopathies.

## 1. Introduction

α-Synuclein (α-syn) is a 140-amino-acid presynaptic protein highly expressed in the vertebrate central nervous system [1,2]. Physiologically, α-syn is localized to synaptic vesicles and is involved in multiple fundamental cellular processes, including the regulation of apoptosis [3,4], participation in SNARE complex assembly [5], mediation of neurotransmitter release [6], and modulation of dopamine homeostasis [7].

Under pathological conditions, α-syn undergoes misfolding and forms insoluble oligomers and amyloid fibrils, which are the primary hallmarks of synucleinopathies such as Parkinson’s disease (PD) and dementia with Lewy bodies (DLB). In DLB, contrary to multiple system atrophy (MSA) where aggregates primarily affect oligodendrocytes, pathological α-syn aggregation and Lewy body deposition occur predominantly in cortical neurons, which is closely associated with progressive cognitive impairment [8,9,10]. The progressive accumulation of these aggregates exerts a toxic gain-of-function. This process directly triggers endoplasmic reticulum stress, neuroinflammation, and apoptosis in late-stage disease. Concurrently, the formation of insoluble inclusions depletes endogenous, soluble α-syn at the presynaptic terminal [11]. Therefore, exploring the loss-of-function of physiological α-syn is equally critical. It provides key insights into the early synaptic dysfunctions and non-motor manifestations of synucleinopathies.

Mood disorders, particularly depression, are highly prevalent in synucleinopathies and often precede the onset of classic motor symptoms, suggesting an intrinsic link between α-syn homeostasis and affective regulation [12]. For instance, clinical studies have shown that serum α-syn levels are significantly elevated in DLB patients comorbid with major depressive disorder compared to healthy individuals [12]. In animal models, affective phenotypes strongly depend on the functional state of α-syn. Transgenic mice overexpressing mutant α-syn frequently develop severe depression-like behaviors in later stages due to progressive neurotoxicity and cellular stress. Conversely, models representing the physiological depletion of the protein, such as α-syn knockout (SYN-KO) mice, present distinct early-stage behavioral profiles. Consistent with early synucleinopathy manifestations, mice lacking synucleins display pronounced hyperactivity and anxiety-like phenotype [13]. Crucially, established SYN-KO models also manifest significant non-motor deficits [13], including reduced responsiveness to rewarding stimuli and impaired learning, which are core features of anhedonia and depression.

Despite these behavioral observations, the precise neural circuit mechanisms linking the loss of α-syn function to depressive-like states remain largely unexplored. Because normal α-syn critically regulates synaptic vesicle dynamics, its absence may disrupt the excitation/inhibition balance within key emotion-regulating hubs, such as the medial prefrontal cortex (mPFC). Given that mPFC GABAergic interneurons play a pivotal role in gating affective networks [14], we hypothesized that the physiological depletion of α-syn could alter inhibitory tone [6]. Therefore, in the present study, we utilized SYN-KO mice to explicitly investigate the association between constitutive α-synuclein depletion and altered stress-coping behaviors. By combining whole-cell patch-clamp electrophysiology and targeted chemogenetic interventions, we aimed to characterize the accompanying changes in medial prefrontal cortex neuronal excitability and determine whether modulating GABAergic interneurons could partially alleviate these affective deficits.

## 2. Results

### 2.1. Identification of α-Syn Gene Knockout Mice

**Figure 1:**
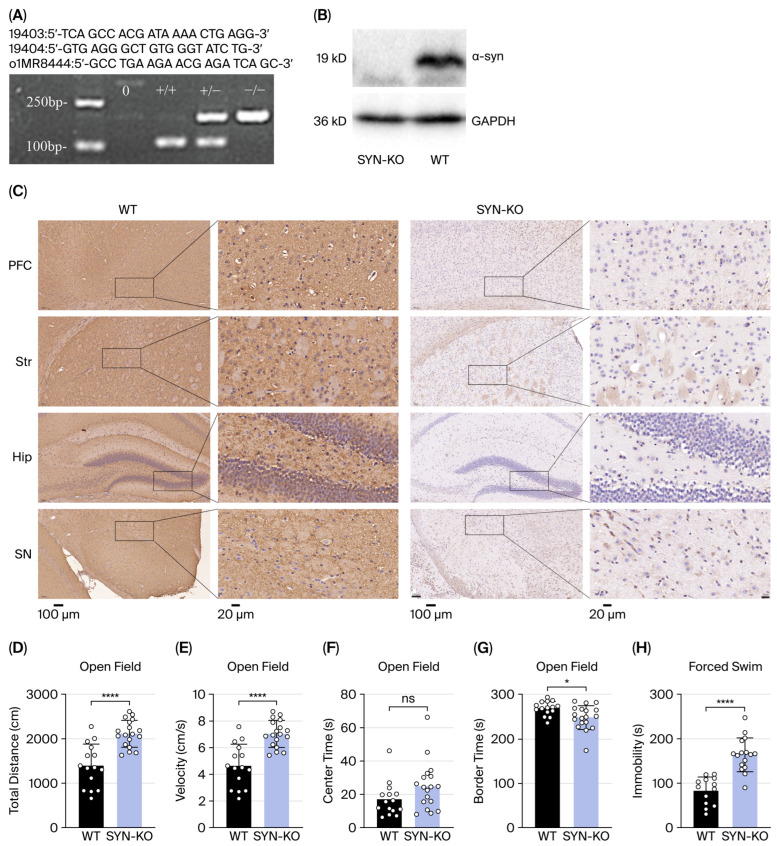
(**A**) Mice genotype identification, wild type (+/+; 105 bp), homozygote (−/−; 192 bp), heterozygote (+/−; 105~192 bp). (**B**,**C**) No α-syn protein expression was seen in the brains of α-syn knockout mice. Bar = 100 μm. (**D**–**H**) Behavioral alterations in α-syn-knockout mice (*n* = 15 mice in WT group, *n* = 18 mice in SYN-KO group): (**D**) total distance moved of mice (eta-squared, η^2^ = 0.4657), (**E**) average moving speed of mice (η^2^ = 0.4659), (**F**) dwell time in the central area (η^2^ = 0.09392), (**G**) dwell time in the border area (η^2^ = 0.1728), and (**H**) forced swimming experiment (η^2^ = 0.5848). The immobility time of mice was within 5 min. Significance levels are indicated as follows: * *p* < 0.05, **** *p* < 0.0001; ns, not significant.

We identified the knockout mice at the gene and protein expression levels. Initially, around 7 days after the mice were born, the tail tips were clipped to extract total DNA from the tail tissue for genotyping. The PCR amplification product of wild-type mice was 105 bp in length; heterozygous knockout mice yielded two PCR product fragments of 105 and 192 bp; and homozygous mice showed a single PCR amplification product of 192 bp (Figure 1A). Subsequently, WB and IHC were used to detect the protein expression level of α-syn in the brains of wild-type and homozygous mice. WB results demonstrated that α-syn protein was not expressed in the brains of α-syn knockout homozygous mice (SYN-KO) (Figure 1B). IHC results revealed that after α-syn knockout, α-syn protein was not expressed in multiple brain regions of the SYN-KO mice, such as in the prefrontal cortex (PFC), striatum (Str), hippocampus (HIP), and substantia nigra (SN) area (Figure 1C).

These results indicate that SYN-KO mice lack α-syn protein expression and are suitable for subsequent studies.

### 2.2. Spontaneous Locomotor Activity Was Increased and Depression-Related Behavioral Despair Was Exacerbated in α-Syn Knockout Mice

We first used the open field test (OFT) to assess psychomotor activity and exploratory reactivity to a novel environment, which are critical parameters for evaluating mood disorders. The open field arena was divided into three zones: the center zone, transition zone, and border zone. The total distance traveled, velocity, time spent in the center zone, and time spent in the border zone were measured for the mice. The results showed that compared with the WT group, the SYN-KO group exhibited a significant increase in total distance traveled (Figure 1D, *p* < 0.0001), an elevated average velocity (Figure 1E, *p* < 0.0001), and reduced time spent in the border zone (Figure 1G, *p* < 0.05). However, there was no significant change in the time spent in the center zone (Figure 1F, *p* > 0.05). These findings indicate that depletion of the α-syn protein induces a state of psychomotor hyperactivity and altered environmental reactivity.

To explicitly evaluate depression-like behavior, we subjected the mice to the forced swim test (FST), a classic behavioral despair paradigm. In this confined space, the animals struggle frantically to escape but are unable to do so. After a period, the animals exhibit a typical despair-like “immobility state”. We observed and recorded the duration of immobility (a sign of despair) within 5 min during the experiment. Notably, the SYN-KO group displayed a significant increase in immobility time compared with the WT group (Figure 1H, *p* < 0.0001). This pronounced transition from hyperlocomotion to acute stress-induced immobility demonstrates that α-syn depletion exacerbates behavioral despair, a core dimension of depression-like behaviors in animal models.

### 2.3. Knockout of α-Syn Is Associated with Enhanced Intrinsic Excitability of mPFC Neurons

To explore the potential mechanisms underlying the behavioral changes in α-syn knockout mice, we detected the excitability of Glut neurons and GABA neurons in mPFC, as well as MSNs in the Str.

**Figure 2:**
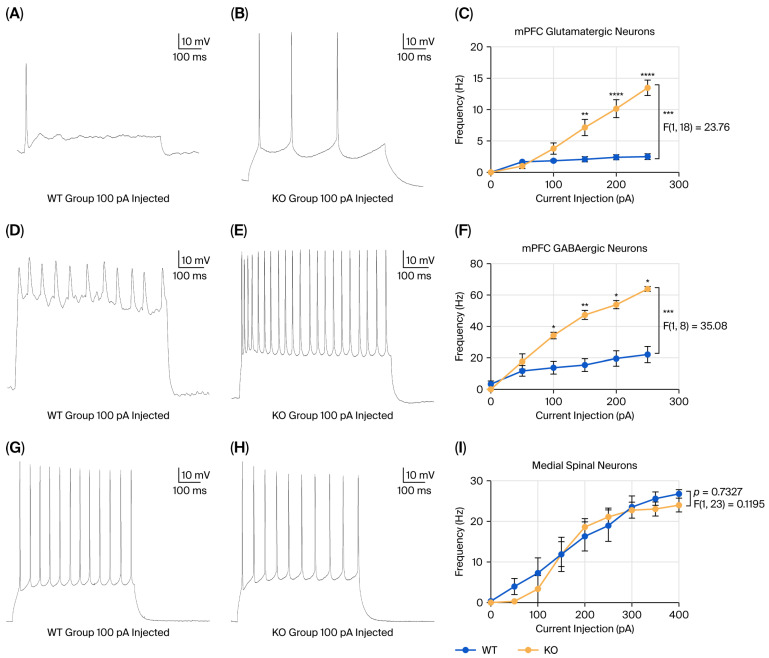
(**A**–**C**) Enhanced intrinsic excitability of mPFC neurons but unaltered MSNs properties after α-syn knockout: (**A**–**C**) Intrinsic excitability of glutamatergic (Glut) neurons in the mPFC: representative action potential traces of Glut neurons in the WT group (**A**) and SYN-KO group (B), and frequency–current (f-I) curves of Glut neurons (**C**). (**D**–**F**) Intrinsic excitability of GABAergic neurons in the mPFC: representative action potential traces in the WT group (**D**) and SYN-KO group (**E**), and f-I curves of GABAergic neurons (**F**). (**G**–**I**) Intrinsic excitability of medium spiny neurons (MSNs) within the striatum (Str): representative action potential traces in the WT group (**G**) and SYN-KO group (**H**), and f-I curves of MSNs (**I**). Sample sizes: Glut neurons (*n* = 9–11 cells from 3 mice per group), GABA neurons (*n* = 6–8 cells from 3 mice per group), and MSNs (*n* = 6–8 cells from 3 mice per group). Statistical significance for f-I curves was determined using a mixed-effects model followed by Bonferroni’s post hoc test. Significance levels are indicated as follows: * *p* < 0.05, ** *p* < 0.01, *** *p* < 0.001, **** *p* < 0.0001 vs. WT group. The main effect was assessed using eta-squared (η^2^), yielding values of 0.569 for Glut neurons, 0.814 for GABAergic neurons, and 0.005 for MSNs.

To assess alterations in action-potential firing of neurons, brain slices were incubated for 1 h in ACSF equilibrated with a gas mixture of 95% O_2_ + 5% CO_2_ prior to placement in the recording chamber. Under the current-clamp configuration, GABA neurons and Glut neurons were subjected to stepwise current stimulation (50 pA increments) over the range of 0–250 pA and action-potential firing was recorded (Figure 2). The number of evoked action potentials varied with the intensity of the applied current. A frequency-current curve was constructed based on the relationship between injected current (current injection, pA) and firing frequency (frequency, Hz) (Figure 2C,F,I).

The results show that within the 150–250 pA current range, action-potential firing frequency of Glut neurons in the mPFC of the SYN-KO group was statistically higher than that of the WT group at each current intensity (Figure 2C, Supplementary Table S3, *p* < 0.001). However, no statistically significant difference in the action-potential firing frequency of Glut neurons was observed between the SYN-KO and WT groups within the 0–100 pA current range (*p* > 0.05).

Further observation of the action-potential firing frequency of GABA neurons in the mPFC at different current intensities revealed that within the 100–250 pA current range, GABA neurons in the mPFC of the SYN-KO group exhibited significantly action-potential firing frequencies than those in the WT group at each current intensity (Figure 2F, Supplementary Table S4, *p* < 0.05). In contrast, no statistically significant difference in the action-potential firing frequency of GABA neurons was observed between the SYN-KO and WT groups within the 0–50 pA current range (*p* > 0.05).

**Figure 3:**
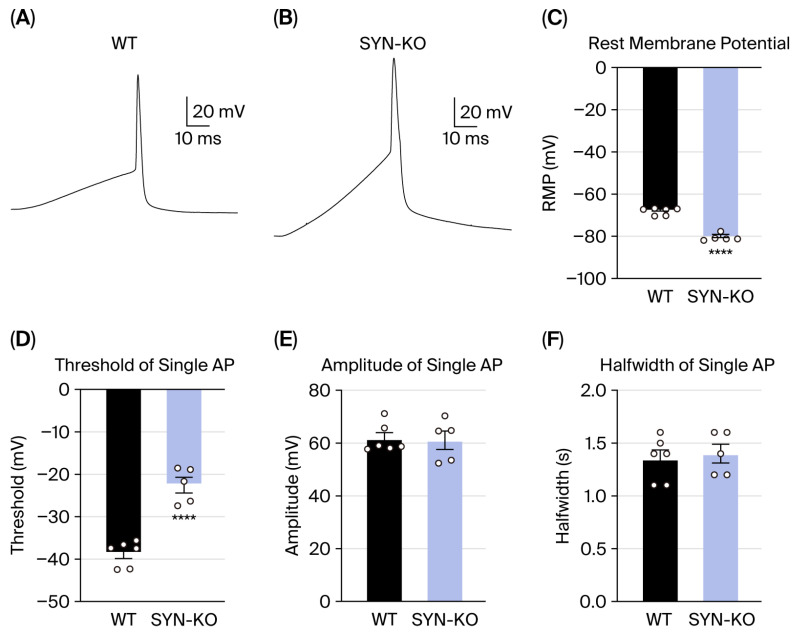
(**A**) Str single MSNs action potentials of mice in the WT group, (**B**) Str single MSNs action potentials of mice in the SYN-KO group, (**C**) rest membrane potentials of Str single MSNs action potentials (η^2^ = 0.9423), (**D**) threshold of single Str MSNs (η^2^ = 0.8626), (**E**) amplitude of Str single MSNs (η^2^ = 0.002738), and (**F**) half-width of Str single MSNs (η^2^ = 0.01786). Significance levels are indicated as follows: **** *p* < 0.0001, *n* = 5–6 cells from 3 mice per group.

In contrast to the cortical neurons, recording of MSNs in the Str revealed that the overall action-potential firing frequency across the 0–400 pA current range was not significantly altered by α-syn depletion (Supplementary Table S5, Figure 2I). However, analysis of individual action potentials of MSNs revealed that compared with the WT group, the absolute value of the resting membrane potential of individual MSNs in the Str of SYN-KO mice was increased, and the absolute value of the threshold of individual action potentials was decreased (Figure 3C,D, *p* < 0.05). Analysis of individual action potential waveforms revealed that both peak amplitude and half-width remained statistically comparable between the two groups (Figure 3E,F; *p* > 0.05).

### 2.4. The Chemogenetic Approach Successfully Suppresses the Excitability of GABA Neurons in the mPFC

Depression pathogenesis is closely associated with dysfunction of neural circuits and abnormal activity of specific neuron populations, among which GABA neurons play a crucial regulatory role [15]. Therefore, we chose to inhibit the excitability of GABA neurons in the prefrontal cortex to explore whether depression-like behaviors in mice could be partially alleviated.

**Figure 4:**
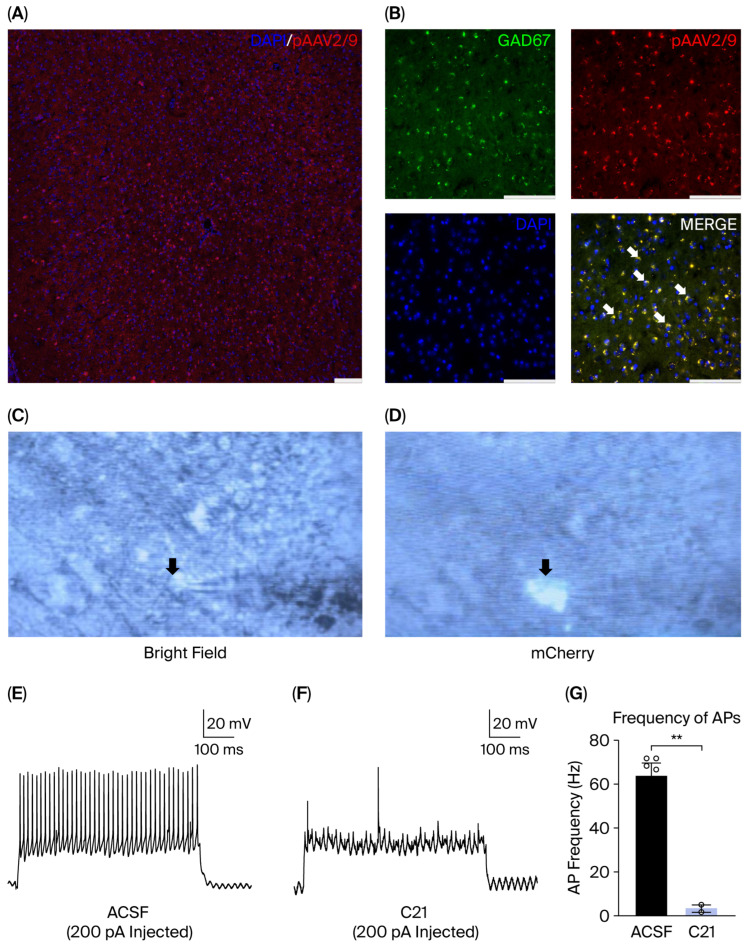
(**A**) GABA neurons in the SYN-KO mouse mPFC. Red fluorescence indicates the expression of pAAV2/9-GAD67-hM4D(Gi)-mCherry- WPRE in the mPFC three weeks after injection. Bar = 100 μm (**B**) Co-localization of GAD67 (green fluorescence) and pAAV2/9 (red fluorescence). White arrows indicate the colocalization of GAD67 and pAAV2/9. Bar = 100 μm. (**C**–**G**) Verification of chemogenetic inhibition of GABA neuronal excitability. (**C**) Schematic diagram of electrode patched cells under bright field. (**D**) Electrode patched cells expressing mCherry. Black arrows indicate that the recorded cells are GABAergic neurons expressing hM4Di.(**E**) Action potentials of GABA cells under 200 pA stimulation. (**F**) Action potential of GABA cells under 200 pA stimulation after administration of C21 (50 μM). (**G**) Administration of C21 (50 μM) effectively inhibited GABA cell excitability (η^2^ = 0.878), Significance levels are indicated as follows: ** *p* < 0.01, *n* = 5 cells from 3 mice per group.

To suppress the excitability of GABA neurons in the mPFC, we stereotactically injected pAAV2/9-GAD67-hM4D(Gi)-mCherry-WPRE (hM4Di) into the brains of SYN-KO mice. Three weeks post-injection, hM4Di was robustly expressed in GABA neurons of the mPFC (Figure 4A,B). Electrophysiological results demonstrated that administration of C21 (50 μM) effectively inhibited the excitability of these GABA neurons (Figure 4E–G).

### 2.5. Chemogenetic Inhibition of mPFC GABA Neurons Partially Mitigates Depression-Related Behavioral Despair

**Figure 5:**
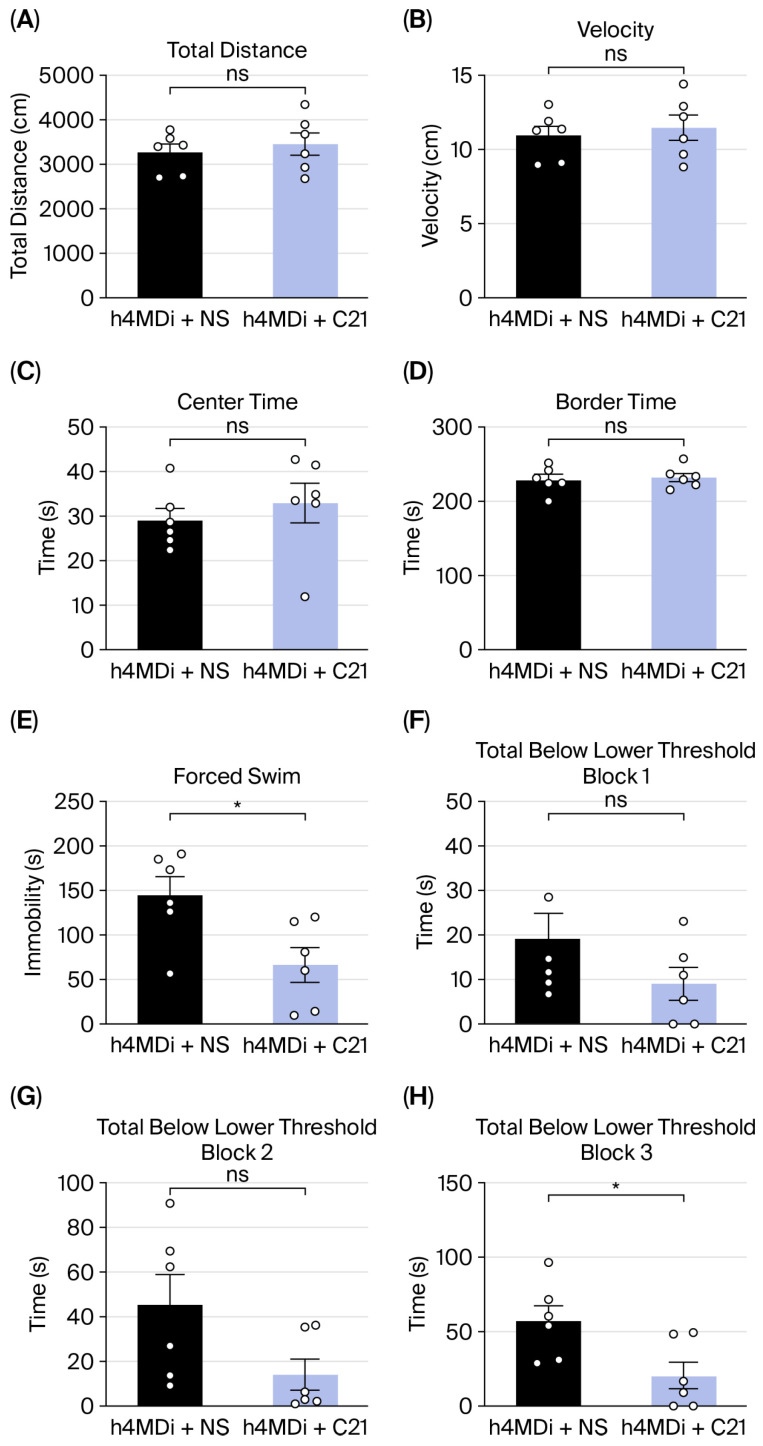
Chemogenetic inhibition of mPFC GABAergic neurons alleviates depression-like behaviors in SYN-KO mice. (**A**–**D**) Open field test results showing no significant differences in total distance (**A**), velocity (**B**), center time (**C**), or border time (**D**) after C21 administration. (**E**) Forced swim test: inhibition of GABA neurons significantly shortened total immobility time. (**F**–**H**) Tail suspension test across three temporal blocks: while showing decreasing trends in Block 1 (**F**) and Block 2 (**G**), chemogenetic inhibition significantly shortened immobility time by Block 3 (**H**) as initial escape struggling subsided. Effect sizes (η^2^) for the comparisons are as follows: A = 0.023, B = 0.033, C = 0.052, D = 0.012, E = 0.434, F = 0.176, G = 0.297, H = 0.419. Significance levels are indicated as follows: * *p* < 0.05, *n* = 6 mice per group; ns, not significant.

Subsequent behavioral profiling aimed to determine the functional impact of dampening mPFC GABAergic excitability in SYN-KO mice. Evaluation within the open field arena revealed that targeted DREADD activation via C21 administration exerted no generalized motor confounds. Specifically, all measured kinematic and spatial parameters—encompassing overall traversal distances, ambulatory speeds, and zonal preferences (center vs. border)—remained statistically indistinguishable from the saline-treated control group (Figure 5A–D, *p* > 0.05). This confirms that the chemogenetic intervention successfully isolated affective circuits without altering basal psychomotor profiles.

However, in the forced swim test, the hM4Di + C21 group exhibited significantly reduced immobility time compared with the control group (Figure 5E, *p* < 0.05). Furthermore, in the tail suspension test, we evaluated the temporal dynamics of immobility across three testing blocks. During the initial highly active phases, where animals inherently exhibit vigorous escape-oriented struggling, the reduction in immobility in the hM4Di + C21 group showed a clear trend but did not reach statistical significance (Figure 5F,G; Block 1: *p* = 0.1742; Block 2: *p* = 0.0672). By block 3, as these initial acute escape responses subsided, a significant reduction in the behavioral despair phenotype emerged, with the hM4Di + C21 group exhibiting reduced immobility time (Figure 5H, *p* < 0.05). Together, these results suggest that dampening the pathological hyperexcitability of GABA neurons in the mPFC partially mitigates the despair dimension of depression-like behaviors in SYN-KO mice without causing locomotor confounds.

## 3. Discussion

In the current study, α-syn knockout mice were utilized to evaluate changes in depression-like behaviors induced by α-syn depletion, as well as to investigate the associated mechanisms. During the open field test (OFT), the knockout animals demonstrated a significant increase in both overall travel distance and movement speed, accompanied by a decrease in the duration spent within the border zone. These alterations reflect baseline psychomotor hyperactivity and altered exploratory reactivity. However, despite this baseline hyperactivity, SYN-KO mice displayed a profound deficit in stress-coping mechanisms during the forced swim test (FST), evidenced by a significant increase in immobility time. Originally developed for screening antidepressant drugs and evaluating treatments in rats and mice [16,17], the FST is now widely used to assess the severity of behavioral impairments induced by factors that may trigger depressive-like states. Our results indicate that α-Syn-KO mice have impaired neural circuits responsible for emotional regulation and stress resilience, resulting in depressive-like behavior.

Historically, mechanistic investigations into synucleinopathies have predominantly focused on pathological α-syn aggregation and the resulting cellular neurotoxicity. Nevertheless, the findings from our current study underscore the necessity of separating this aggregation-mediated toxicity from the primary, physiological loss-of-function of the protein. In pathological states, the progressive accumulation of intracellular inclusions exerts a toxic gain-of-function while concurrently depleting endogenous, soluble α-syn at presynaptic terminals. Previous studies utilizing overexpression of α-syn in the hippocampus replicates depressive-like phenotypes, whereas the genetic deletion of α-syn enhances resistance to chronic stress [18]. Depression may be associated with an increased risk of dementia [19]. A previous study measuring serum levels in patients with DLB found that serum α-syn concentrations were significantly higher in those with comorbid major depressive disorder than in healthy individuals [12]. However, our findings in the SYN-KO model reveal that the complete physiological absence of α-syn also results in basal behavioral despair. Rather than a contradiction, this bidirectional sensitivity indicates that affective neural networks rely heavily on strict α-syn homeostasis. Endogenous α-syn plays a vital physiological role in regulating synaptic vesicle dynamics and neurotransmitter release. Our data suggest that the loss of these physiological synaptic functions is fully sufficient to disrupt mood-regulating circuits. This circuit disruption can occur well victory to the onset of any aggregation-mediated toxicity, or it may exist entirely independent of such pathological processes. To investigate the mechanisms underlying behavioral alterations in α-syn knockout mice, we performed whole-cell patch-clamp recordings to assess changes in the intrinsic excitability of GABA neurons in the mPFC. Our results showed that GABA neurons in the mPFC of α-syn knockout mice exhibited enhanced excitability, reflected by an increased action-potential firing frequency. Specifically, at a given current injection amplitude, these neurons generated action potentials more readily than controls, indicating elevated excitability. Given that the excitability of GABA neurons has been linked to depression [14,20,21,22], we hypothesized that increased excitability of mPFC GABA neurons may contribute to the development of depression-like behavior in α-syn knockout mice.

Next, to determine the causal relationship between this GABAergic hyperexcitability and the behavioral phenotype, we employed a chemogenetic (DREADD) approach using the inhibitory receptor hM4Di activated by the specific ligand C21. Chemogenetic techniques enable targeted regulation of neuronal activity in specific cell populations and neural circuits through the administration of exogenous ligands that activate designer receptors exclusively activated by designer drugs (DREADDs) [23,24]. In behavioral neuroscience, these tools are widely used in preclinical models to investigate the roles of specific neural circuits and neuronal subpopulations. Commonly employed DREADDs include Gq (hM3Dq), Gi (hM4Di), and Gs (rM3Ds), among which Gi (hM4Di) exerts an inhibitory effect. Traditionally, clozapine-N-oxide (CNO) has been used to activate Gi (hM4Di) [25]; however, because CNO can be partially converted to clozapine in the brain, novel compound C21 has been developed as an alternative in recent years [26,27]. Previous evidence establishes that dysfunction in GABAergic interneurons, particularly aberrant excitability, is a core pathogenic factor in depression. Pathological hyperactivity of mPFC GABAergic neurons imposes excessive feedforward and feedback inhibition on local neural circuits, thereby suppressing the excitatory output of emotion-regulating pathways [15,28]. In our study, appropriately suppressing these hyperactive GABAergic neurons via C21 administration rapidly alleviated the depression-like phenotype (immobility in the FST) in SYN-KO mice without altering baseline locomotor activity. This targeted rescue provides strong functional evidence that the hyperexcitability of mPFC GABAergic neurons specifically mediates the behavioral despair induced by α-syn depletion.

The present study has several limitations that warrant consideration. First, whole-cell recordings revealed hyperexcitability in both glutamatergic and GABAergic neurons within the mPFC of SYN-KO mice. However, our chemogenetic rescue strategy selectively targeted the GABAergic population. This design was based on the well-established hierarchical microcircuitry of the mPFC. Anatomically, the mPFC neural network consists predominantly of excitatory glutamatergic pyramidal neurons, while GABAergic interneurons constitute only 10 to 20 percent of the neuronal population [29]. Despite their smaller proportion, these GABAergic interneurons act as the critical brake system of the brain. They directly regulate the excitability of glutamatergic pyramidal neurons by targeting specific subcellular domains [30]. This precise targeting allows them to govern local network dynamics, control firing rates, and maintain the excitation and inhibition balance within the cortex [31]. Relieving this overactive local inhibition is demonstrated to yield significant antidepressant outcomes. Because glutamatergic neurons are predominantly responsible for long-distance communication to subcortical emotion-regulating regions, their concurrent hyperexcitability likely contributes to the complex affective alterations following α-syn depletion [30,31]. We did not perform a parallel chemogenetic rescue targeting mPFC glutamatergic neurons, and future studies employing dual-vector systems are required to fully dissect the interactive contributions of these distinct neural types. Second, the chemogenetic inhibition of mPFC GABAergic neurons was performed exclusively in SYN-KO mice as a targeted rescue paradigm. While recent pharmacological validations confirm that the DREADD agonist C21 exhibits high target specificity and lacks off-target behavioral effects at the dosage used, the absence of a wild-type (WT) hM4Di cohort limits our ability to evaluate the effects of this specific GABAergic manipulation entirely independent of the α-syn knockout background. Finally, our behavioral evaluation primarily relied on the OFT and FST/TST paradigms. While these effectively capture baseline hyperactivity and acute stress-induced despair, future studies incorporating additional assays, such as the sucrose preference test or novelty-suppressed feeding test, are necessary to comprehensively evaluate the anhedonic and motivational dimensions of the SYN-KO phenotype.

## 4. Materials and Methods

### 4.1. Animals

Male WT C57BL/6 mice (8–12 weeks old) were purchased from Beijing Huafukang Biotechnology Co., Ltd. Gene knockout mice (SYN-KO) were kindly provided by Professor Bin Xu [32] from China Medical University, and embryo resuscitation was performed at the Genetic Center of our institute. All animals were housed in a barrier animal facility with a relative humidity of 50 ± 5%, a temperature of 24 ± 2 °C, and a 12/12 h light/dark cycle. Food and water were available ad libitum. All experimental procedures were approved by the Institutional Animal Care and Use Committee (IACUC) of the Institute of Laboratory Animal Science, Chinese Academy of Medical Sciences, and Peking Union Medical College. All WT and SYN-KO mice were littermates and were group-housed.

### 4.2. Viral Constructs and Surgery

Adeno-associated viruses pAAV2/9-GAD67-hM4D(Gi)-mCherry-WPRE (hM4Di, ≥4.09 × 10^12^ v.g./mL) were obtained from OBiO (Shanghai, China). Animals (SYN-KO, 20–24 weeks old) were fasted for 24 h before surgery. Under isoflurane anesthesia (induced at 2 L/min and maintained at 1.5 L/min), mice were bilaterally injected with the inhibitory hM4Di vector into the mPFC (AP: 1.9 mm, ML: ±0.35 mm, DV: −3.0 mm). These specific stereotaxic coordinates were selected based on established protocols for accurately targeting the mouse mPFC [33]. A volume of 0.5 μL per hemisphere was delivered at a rate of 250 nL/min, and the subjects were allowed a 3-week recovery period prior to further studies. Histological verification of the viral injection site in the mPFC in Supplementary Figure S1.

### 4.3. Drug Administration

DREADD agonist 21 (compound 21, C21) was administered alone (APExBIO, 2 mg/kg i.p.). C21 has excellent bioavailability, pharmacokinetic properties, and brain penetrability [34]. It has been reported that the compound does not undergo back-metabolism to clozapine. Behavioral tests were performed 40 min after C21 injection (hM4Di + C21, *n* = 6). An equivalent volume of physiological saline was administered via intraperitoneal delivery to the mice of control group (hM4Di + NS, *n* = 6).

### 4.4. Behavioral Studies

To ensure unbiased data acquisition, all behavioral paradigms were executed by researchers strictly blinded to animal genotypes and experimental group assignments. Prior to the commencement of data collection, male subjects (WT and SYN-KO groups, strictly aged 20–24 weeks) underwent a 7-day acclimatization phase. During this period, mice were habituated to the testing environment for 60 min daily before any scheduled procedures. To minimize circadian variations, all assays were exclusively performed during the daytime cycle (between 10:00 and 15:00). Testing sequences were fully counterbalanced, and subjects were assigned to respective treatment arms utilizing a randomized block design.

Open field test (OFT): Basal psychomotor profiles and exploratory reactivity to novel surroundings were quantified using square enclosures (50 cm × 50 cm × 50 cm) under ambient lighting conditions. Individual mice were introduced into the center of the arena and permitted to navigate freely for a 5 min tracking window. Locomotor parameters (overall ambulatory distance and average velocity) and spatial habitation patterns (time spent in the central versus peripheral border zones) were continuously captured and processed utilizing the EthoVision XT 2.3 tracking system (Noldus Information Technology, Leesburg, VA, USA) [35].

Forced swim test (FST): Acute stress-coping strategies and behavioral despair were evaluated via the FST. Mice were individually placed into vertical, transparent cylinders (7.5 cm internal diameter, 25 cm height) filled with water maintained at 23 ± 1 °C. The water depth was calibrated to leave a 7 cm clearance from the cylinder rim, preventing escape while ensuring animals could not touch the bottom. Conducted under 300 lx ambient illumination, the cumulative duration of immobility—characterized by the cessation of active escape behaviors—was recorded over a continuous 5 min observation period.

Tail suspension test (TST): As a complementary evaluation of depressive-like states, subjects were assessed using a custom white acrylic apparatus (30 cm × 30 cm × 40 cm) featuring an open front for uninterrupted video monitoring. Under controlled illumination (250 lx), each mouse was secured by the distal portion of its tail and suspended approximately 2 cm above the chamber floor. The total testing duration spanned 6 min, which was subsequently subdivided into three distinct 2 min epochs for dynamic temporal analysis. In this paradigm, “immobility” was strictly defined as the complete cessation of movement and struggling lasting for a minimum of 1 continuous second.

### 4.5. Genotype Identification by PCR

Genomic DNA was extracted from mouse tails for genotype identification. PCR was performed using the reaction mixture (Supplementary Table S1), primer sequences (Figure 1A), and thermal cycling conditions (Supplementary Table S2). The resulting PCR amplicons were resolved via electrophoresis on a 1% agarose matrix (120 V, 30 min) and subsequently detected using a 365 nm ultraviolet transilluminator. Uncropped captures are accessible in Supplementary Figure S2.

### 4.6. Protein Extraction and BCA Assay

To isolate total cellular proteins, murine brain specimens were homogenized using ice-cold RIPA lysis solution. To prevent endogenous proteolytic degradation and preserve structural integrity, the extraction matrix was fortified immediately prior to use with a dual-blockade system comprising protease and phosphatase inhibitors (Complete™ and PhosStop™ cocktails, Roche, Basel, Switzerland).

Samples were weighed, and lysis buffer was added (1000 μL:100 g of pre-cooled lysis buffer). The mixture was kept on ice. Magnetic beads were placed into a 1.5 mL EP tube, which was then transferred to an automatic sample grinder (50 Hz, 30 s, Shanghai Jinxin Industrial Development Co., Ltd., Shanghai, China). If the tissue lysis was insufficient, this step was repeated.

After clarification by centrifugation (12,000× *g*, 5 min, 4 °C), the resulting soluble supernatant was collected. Protein yields were subsequently quantified utilizing the Pierce™ BCA Protein Assay Kit (Thermo Fisher Scientific, Waltham, MA, USA). Briefly, sample aliquots were reacted with the BCA working reagent for 30 min at 37 °C, after which the colorimetric absorbance was recorded at 562 nm via a NanoDrop 2000 spectrophotometer (Thermo Fisher Scientific, Waltham, MA, USA).

### 4.7. Western Blot Analysis

Target protein expression was profiled using a primary rabbit anti-α-synuclein probe (1:1000, #ab138501; Abcam, Cambridge, UK), which was subsequently recognized by an HRP-conjugated goat anti-rabbit secondary IgG (1:10,000, #ab6721; Abcam). Chemiluminescent signals were developed via the Clarity Western ECL system (Bio-Rad, Hercules, CA, USA). To guarantee uniform sample loading and enable accurate densitometric normalization, GAPDH (1:1000, #5174; Cell Signaling Technology, Danvers, MA, USA) was utilized as the endogenous reference. Uncropped raw blot captures are accessible in Supplementary Figure S3.

### 4.8. Immunohistochemistry

Following deep isoflurane-induced anesthesia, murine subjects underwent transcardial clearance with physiological saline, immediately followed by in situ fixation using chilled 4% paraformaldehyde (PFA, formulated in 0.01 M PBS). Intact brains were harvested, subjected to a 24 h postfixation in 4% PFA, and subsequently processed into formalin-fixed paraffin-embedded (FFPE) blocks. Microtome-sectioned coronal slices (5 μm thickness) were treated with 0.3% H_2_O_2_ for 10 min to quench endogenous peroxidase activity. Following buffer rinses, non-specific binding sites were masked via a 1 h incubation in 10% normal goat serum. Tissue probing involved an overnight exposure at 4 °C to the primary anti-α-synuclein antibody (1:500, #ab138501; Abcam), succeeded by a 1 h ambient-temperature incubation with the corresponding HRP-linked secondary antibody (1:10,000, #ab6721; Abcam). Antibody labeling was visualized using 0.5 mg/mL 3,3′-diaminobenzidine (DAB). Coverslips were mounted with neutral resin mounting medium using a Leica automatic coverslipper (Wetzlar, Germany).

### 4.9. Viral Infection Efficiency and Immunofluorescence

Mice were deeply anesthetized with isoflurane (inhalation) and transcardially perfused as previously described. Brains were then coronally sectioned at a thickness of 30 µm, with sections containing the mPFC. Immunofluorescence staining for GAD67 was performed using a primary antibody (rabbit anti-GAD67, #ab213508, 1:50, Abcam) and a secondary antibody (AlexaFluor^®^ 488 goat anti-rabbit, #ab150077, 1:1000, Abcam). Viral expression and injection sites were observed under a fluorescence microscope (Leica, Wetzlar, Germany).

### 4.10. Electrophysiological Recordings

GABAergic (GABA) neurons and glutamatergic (Glut) neurons in the mPFC, as well as medium spiny neurons (MSNs) in the striatum (Str), were recorded. Coronal slices (250 μm) containing the mPFC were prepared from male SYN-KO and WT mice for whole-cell patch-clamp recording, as previously described [36]. Glass electrodes with a resistance of 4–6 MΩ were prepared, and the holding potential was clamped at −70 mV. Prior to current-clamp recording, series resistance was monitored and canceled using a bridge circuit, and pipette capacitance was compensated. After establishing the whole-cell current-clamp configuration, cells were allowed 10 min to stabilize their resting membrane potentials. A current-step protocol (from −200 to 400 pA in 50 pA increments; inter-pulse interval: 15 s) [37] was then applied for at least three runs. Only neurons with stable resting membrane potentials and consistent evoked action-potential firing were included in the analysis.

To specifically evaluate the chemogenetic suppression of mPFC GABAergic interneurons, whole-cell recordings were conducted on acute coronal sections derived from 20- to 24-week-old male SYN-KO mice previously injected with the hM4D(Gi) viral vector. Successfully transduced interneurons were positively identified via mCherry fluorescence under specific optical filters. To trigger inhibition, the DREADD agonist C21 (50 μM) was bath-applied through the circulating artificial cerebrospinal fluid (ACSF), allowing for real-time monitoring of altered neuronal excitability. Stringent quality control criteria were enforced during this phase: only recordings maintaining a resting potential more negative than −55 mV and exhibiting less than 20% fluctuation in access resistance were retained for downstream evaluation.

### 4.11. Statistical Analysis

Quantitative analyses were executed via SPSS version 23.0 software, with data visualization performed utilizing GraphPad Prism version 9.5.1 software. For baseline two-group comparisons, independent-samples t-tests were applied, provided the datasets met the assumptions of normal distribution.

To properly address the hierarchical structure of the electrophysiological data—specifically, multiple cells sampled within individual animals (nested data)—we employed a mixed-effects modeling approach to analyze the frequency-current (f-I) curves. This was followed by Bonferroni post hoc corrections for multiple comparisons. All descriptive statistics are presented as the mean ± standard deviation (mean ± SD), with the threshold for statistical significance predefined at *p* < 0.05.

## 5. Conclusions

In summary, the physiological depletion of endogenous α-synuclein is associated with depression-like behaviors and increased mPFC neuronal excitability. Chemogenetic suppression of mPFC GAD67-positive interneurons partially reduces immobility in SYN-KO mice, implicating prefrontal inhibitory circuit dysregulation in these behavioral alterations. These findings underscore the importance of α-syn homeostasis in modulating cortical microcircuits and provide novel insights into the early non-motor manifestations of synucleinopathies.

## Data Availability

The original contributions presented in this study are included in the article/Supplementary Material. Further inquiries can be directed to the corresponding author.

## Supplementary Materials

The following supporting information can be downloaded at: https://www.mdpi.com/article/10.3390/ijms27125235/s1.
