# Supplementary material for: Knockout of α-Synuclein Is Associated with Depression-like Behaviors by Altered Excitability of Medial Prefrontal Cortex Neurons in Mice
Source: Int J Mol Sci. 2026 Jun 9;27(12):5235. doi: 10.3390/ijms27125235 (PMC13299995; doi:10.3390/ijms27125235)

Supplementary

Table S1. PCR reaction system for genotyping of α-syn knockout mice.

Component	Volume (μL)
2× M-PCR OPTI™ Mix	10
Primer 19403 (10 μM)	0.5
Primer 19404 (10 μM)	0.5
Primer oLMR884 (10 μM)	0.5
Template DNA	1
ddH2O	6
Total volume	20

Table S2. PCR reaction conditions for α-syn knockout genotyping.

Step	Temperature	Time	
Pre-denaturation	94 °C	2 min	
Denaturation	94 °C	30 s	35 cycles
Annealing	58 °C	30 s	
Extension	72 °C	40 s	
Final extension	72 °C	10 min	
	4 °C	∞	

Table S3. Action-potential firing frequencies of glutamatergic neurons within the mPFC at different current injection steps.

Injected current (pA)	WT(Hz) (N=9)	SYN-KO(Hz) (N=11)	Adjusted *P*-Value	Significance	DF
0	0	0	>0.999	ns	91.00
50	1.667 ± 0	1.000 ± 0.444	>0.999	ns	91.00
100	1.852 ± 0.185	3.788 ± 0.901	0.6853	ns	91.00
150	2.083 ± 0.417	7.167 ± 1.316	0.0075	**	91.00
200	2.407 ± 0.404	10.150 ± 1.447	<0.0001	****	91.00
250	2.500 ± 0.481	13.48 ± 1.240	<0.0001	****	91.00

Note: Results are presented as mean ± SEM. Statistical significance was determined using a mixed-effects model followed by Bonferroni's post hoc multiple comparisons test. ns, not significant; ** $P < 0.01$; **** $P < 0.0001$ compared to the WT group.

Table S4. Action-potential firing frequencies of GABAergic neurons within the mPFC at different current injection steps.

Injected Current (pA)	WT (Hz) (N = 8)	SYN-KO (Hz) (N = 6)	Adjusted *P*-Value	Significance	DF
0	3.333 ± 1.925	0	>0.999	ns	3.000
50	11.670 ± 3.402	17.670 ± 4.876	>0.999	ns	6.719
100	13.750 ± 4.047	34.170 ± 2.053	0.0478	*	4.561
150	15.420 ± 4.104	47.330 ± 2.867	0.0054	**	5.637
200	19.580 ± 4.877	53.890 ± 2.606	0.0116	*	4.726
250	22.080 ± 5.154	64.000 ± 1.716	0.0129	*	3.668

Note: Results are presented as mean ± SEM. Statistical significance was determined using a mixed-effects model followed by Bonferroni's post hoc multiple comparisons test. ns, not significant; * $P < 0.05$; ** $P < 0.01$ compared to the WT group.

Table S5. Action-potential firing frequencies of medium spiny neurons (MSNs) within the striatum (Str) at different current injection steps.

Injected Current (pA)	WT (Hz) (N = 8)	SYN-KO (Hz) (N = 6)	Adjusted *P*-Value	Significance	DF
0	0.357 ± 0.357	0	>0.999	ns	13.00
50	3.958 ± 1.990	0.278 ± 0.278	0.9733	ns	7.271
100	7.292 ± 3.726	3.333 ± 3.333	>0.999	ns	11.96
150	11.880 ± 4.231	11.940 ± 3.056	>0.999	ns	11.74
200	16.310 ± 3.577	18.640 ± 2.045	>0.999	ns	20.10
250	18.960 ± 3.870	21.110 ± 2.180	>0.999	ns	10.65
300	23.540 ± 2.754	22.780 ± 1.956	>0.999	ns	11.68
350	25.620 ± 1.665	23.060 ± 1.744	>0.999	ns	11.47
400	26.790 ± 1.055	24.000 ± 1.689	>0.999	ns	15.74

Note: Results are presented as mean ± SEM. Statistical significance was determined using a mixed-effects model followed by Bonferroni's post hoc multiple comparisons test. ns, not significant compared to the WT group.

Figure S1. Histological verification of the viral injection site in the mPFC.

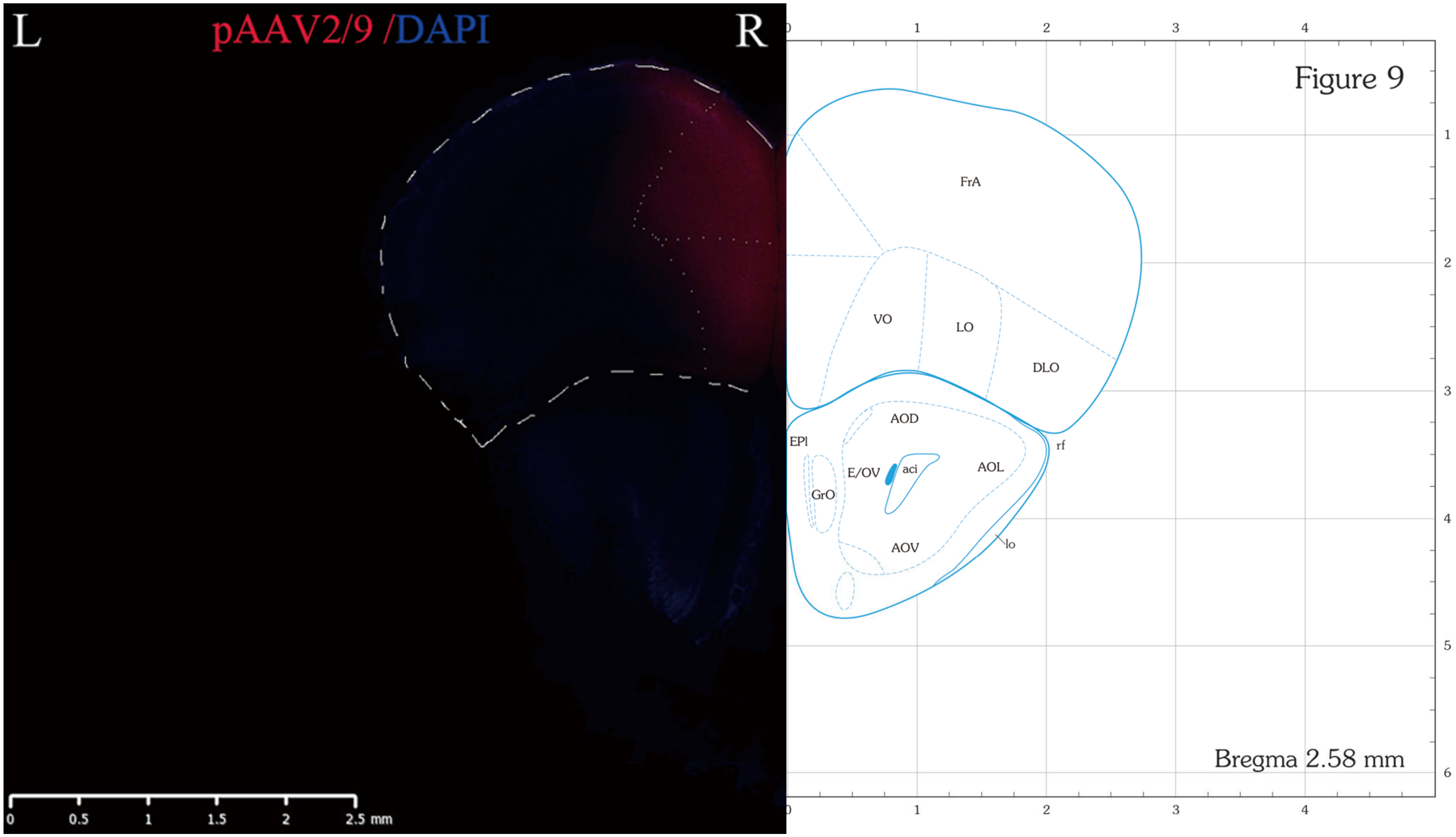

Figure S2. Original images of PCR amplicons were resolved via electrophoresis on a 1% agarose matrix of Figure 1A.

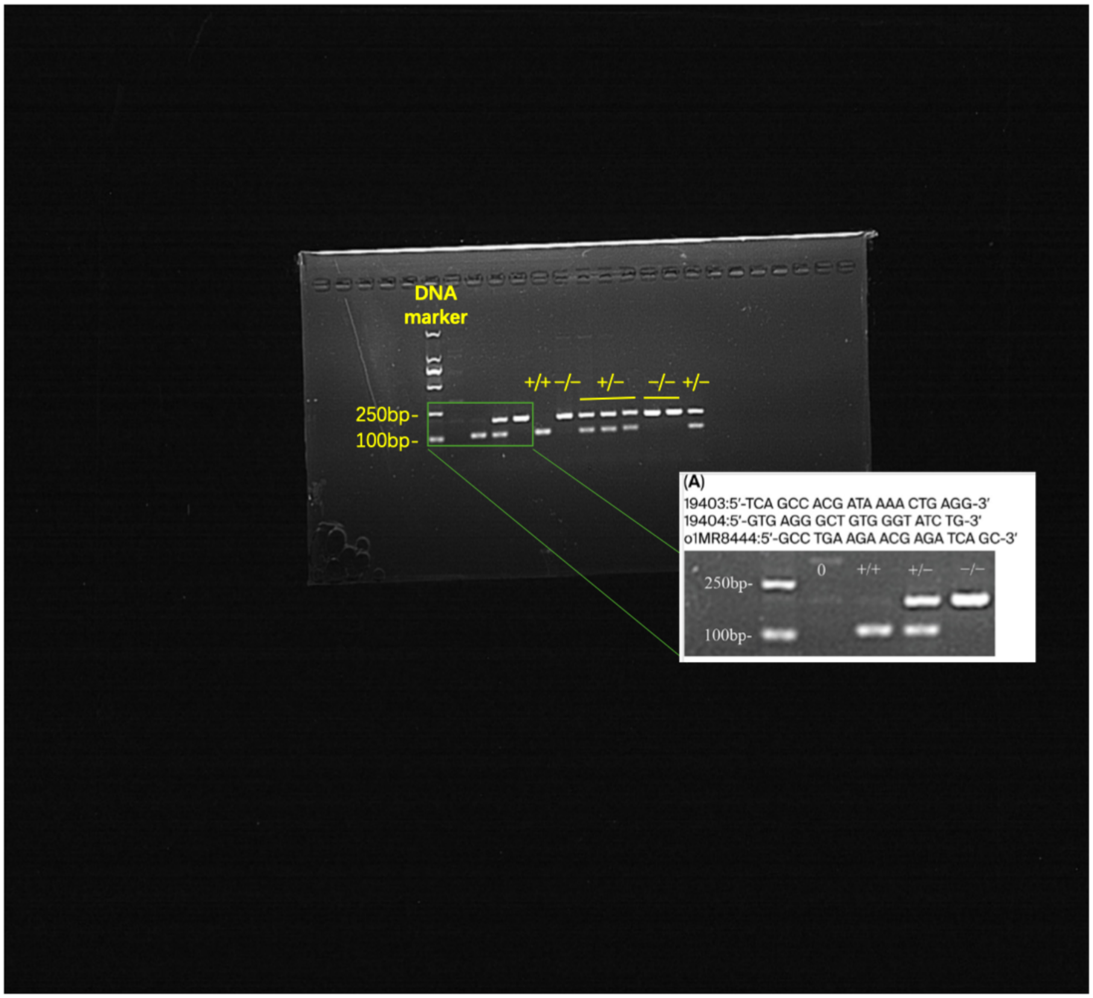

Figure S3. Original Western blot images of Figure 1B.

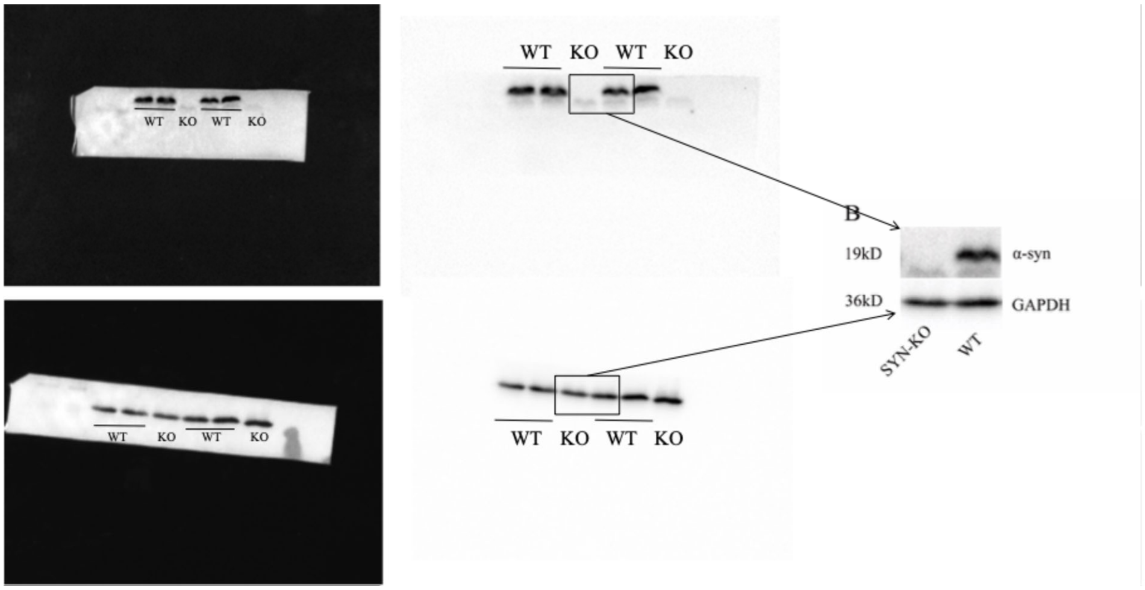

Supplement: Supplementary file 1 [file ijms-27-05235-s001.zip › ijms-4314659-supplementary.pdf]
